# Analysis of the Healthcare System in Romania: A Brief Review

**DOI:** 10.3390/healthcare11142069

**Published:** 2023-07-19

**Authors:** Ion Petre, Flavia Barna, Daniela Gurgus, Laurentiu Cezar Tomescu, Adrian Apostol, Izabella Petre, Cristian Furau, Miruna Lucia Năchescu, Anca Bordianu

**Affiliations:** 1 Department of Functional Sciences, Medical Informatics and Biostatistics Discipline, “Victor Babeș” University of Medicine and Pharmacy, 300041 Timisoara, Romania; petre.ion@umft.ro; 2 Doctoral School, Faculty of Economics and Business Administration, West University of Timisoara, 16 J.H. Pestalozzi Street, 300115 Timisoara, Romania; 3 Department of Finance, Faculty of Economics and Business Administration, West University of Timișoara, 16 Pestalozzi Street, 300115 Timisoara, Romania; flavia.barna@e-uvt.ro; 4 Department of Balneology, Medical Recovery and Rheumatology, Centre for Preventive Medicine, “Victor Babes” University of Medicine and Pharmacy, Eftimie Murgu Sq. Nr. 2, 300041 Timisoara, Romania; 5 Faculty of Medicine, Ovidius University of Constanta, 900527 Constanta, Romania; tomescu.cezar.laurentiu@gmail.com; 6 Department VII of Internal Medicine, “Victor Babeș” University of Medicine and Pharmacy, 300041 Timisoara, Romania; dr_apostol@yahoo.co.uk; 7 Department XII, Discipline of Obstetrics and Gynecology III, “Victor Babeș” University of Medicine and Pharmacy, 300041 Timisoara, Romania; petre.izabella@umft.ro; 8 Department of Obstetrics and Gynecology, “Vasile Goldiş” Western University of Arad, 310025 Arad, Romania; cristianfurau@gmail.com; 9 Finance Department, Faculty of Economics and Business Administration, West University of Timisoara, 300223 Timisoara, Romania; miruna.nachescu@e-uvt.ro; 10 Department of Plastic Surgery and Reconstructive Microsurgery Bagdasar-Arseni, Emergency Hospital Bucharest, University of Medicine and Pharmacy “Carol Davila”, 010825 Bucharest, Romania; anca.bordianu@gmail.com

**Keywords:** healthcare system, Romania, analysis, infrastructure, financing, challenges, reforms

## Abstract

This manuscript provides a brief review and analysis of the healthcare system in Romania. This study aims to comprehensively analyse the healthcare system in Romania, evaluating its strengths, weaknesses, and impact on the population’s access to quality healthcare services. Within the framework of the Romanian healthcare system, a multitude of pressing challenges endure. These encompass insufficient funding, shortages of medical personnel, and ineffectiveness in the provisioning of services. These impediments substantially hinder the accessibility of healthcare services, particularly in outlying and pastoral regions, thereby rendering the system susceptible and underserving certain demographics. Our investigation presents three hypotheses. The opening conjecture proposes that inadequate funding has a negative impact on the availability and standard of healthcare facilities in Romania. In addition, another hypothesis assumes that insufficient medical staff plays a considerable role in inequalities in access to and delivery of healthcare. Moreover, the existence of inadequacies in service provision serves as a significant barrier, obstructing the timely and efficient delivery of healthcare to those who need it. Our research encompasses a comprehensive analysis of key aspects of the Romanian healthcare system, ranging from healthcare infrastructure and financing mechanisms to service delivery and healthcare outcomes. Through a blend of qualitative and quantitative data sources, including government reports, academic studies, and statistical data, we have endeavoured to provide an in-depth evaluation. The analysis encompasses various aspects, including healthcare infrastructure, financing mechanisms, service delivery, and healthcare outcomes. Romania has a mixed healthcare system with both public and private providers. The primary level of care is delivered by family doctors, while hospitals and specialised medical centres provide secondary and tertiary care services. This research underlines the criticality of significant alterations being implemented in the healthcare system of Romania to address the issues arising from insufficient funding, a shortage of medical personnel, and shortcomings in service delivery. It is vital to tackle the obstacles presented by insufficient funding, the dearth of healthcare staff, and inadequacies in service delivery to attain impartial and reachable healthcare. By implementing these essential transformations, Romania can pave the way towards a healthcare system that efficaciously caters to the diverse requirements of its populace and guarantees the provision of prompt and superior healthcare services.

## 1. Introduction

Romania is a country in the southern part of Central Europe, bordering Eastern Europe and the Balkan Peninsula, in the northern hemisphere of the globe [1]. Romania is bordered by Ukraine, Moldova, Bulgaria, Serbia, and Hungary.

Since 1 January 2007, Romania has been a member state of the European Union. With a surface area of 238,397 km^2^, Romania constitutes 4.8% of Europe and 5.4% of the European Union [1]. The estimated population of Romania as of 1 January 2022, is 21,980,534.

Romania is an upper-middle-income country with an economy that is the 13th largest in the European Union and the 49th largest in the world [2]. The Romanian economy has undergone significant changes in the past few decades, transitioning from a centrally planned economy to a market-oriented one. Romania has a mixed economy that is dominated by the service sector, which accounts for approximately 60% of the country’s GDP [2]. The industrial sector is the second-largest contributor to the economy, accounting for around 20% of GDP, while agriculture contributes approximately 4.2% of GDP [3]. Over the past two decades, Romania has impressively grown and prospered. Since 2010, the country’s economy has grown at one of the fastest rates in the European Union. Romania’s gross domestic product (GDP) is estimated at around USD 284.9 billion, with a GDP per capita of USD 14,872 [2]. The unemployment rate in Romania has been steadily declining, reaching a record low of 3.9% in 2019. Despite these efforts, Romania still faces some challenges, including a high level of income inequality and a lack of access to healthcare and education in some areas. The country also faces environmental challenges, including air and water pollution.

The findings of this manuscript can inform policymakers, healthcare professionals, and researchers about the current state of the Romanian healthcare system. The identification of key areas requiring improvement, such as infrastructure, regional disparities, the healthcare workforce, and funding, offers valuable insights for policy interventions and strategic planning. Moreover, the analysis highlights successful aspects of the healthcare system that can serve as potential models or best practices for other countries facing similar challenges.

This manuscript’s contribution lies in its comprehensive and scientifically grounded examination of the Romanian healthcare system, providing actionable insights and facilitating evidence-based decision-making in healthcare policy and practice.

## 2. Healthcare Infrastructure in Romania

The latest report of the Romanian National Institute of Statistics shows that there are more than 65,000 health units operating in the country, with 53,000 in urban areas and 12,000 in rural areas [4]. The number of hospitals in the country is 543 (488 in urban areas and 55 in rural areas), but there are also 160 other hospital-like establishments, such as medical centres, diagnostic centres, health centres, and other hospital-like medical establishments providing day hospital services only (not inpatient continuous admission) [5]. Of the total number of hospitals and hospital-like establishments, 49.8% were large establishments with more than 100 beds for continuous or day hospitalisation, and 38.7% were small establishments with less than 50 beds [5]. The total number of continuous inpatient beds available in hospitals is 135,085. According to the specialty for which hospital beds were allocated; psychiatry (12.0%); surgery (10.4%); internal medicine (8.9%); obstetrics, gynaecology, and rehabilitation; physical medicine; and balneology (6.3% each); and pneumology (6.2%) received the most hospital beds [5]. Following that are paediatrics (5.4%), infectious diseases (4.5%), cardiology and ATI (4.3% each), neurology (4.0%), neonatology (3.4%), orthopaedics and traumatology (3.2%), and oncology (3.1%).

Romania faces unequal access to healthcare between settings. In urban areas, there are 90.9% of the total number of hospitals and hospital-like establishments, 92.3% of the total number of specialist outpatient clinics and hospital-integrated outpatient clinics, 97.3% of the total number of medical dispensaries, 97.8% of the total number of dialysis centres, 98.5% of the total number of specialist medical centres, as well as nine out of eleven spa sanatoriums, all mental health centres, blood transfusion centres, and TB sanatoria. Additionally, urban areas have 60.5% of independent family medicine practices, 85.3% of independent general practice practices, 85.5% of independent dental practices, 94.9% of ‘‘other types of medical practices’’ (occupational medicine practices, company practices, medical expertise, and rehabilitation practices, etc.), 95.2% of independent specialist medical practices, 98.9% of school medical practices, 99.8% of school dental practices, all student medical and dental practices, 61.8% of pharmacies, pharmacy outlets, and drugstores, 95.4% of dental laboratories, 95.5% of medical laboratories, and 97.5% of ambulance and patient transport [5].

There are 366,821 health professionals working in Romania’s healthcare system. Of these, 36.9% are highly qualified health professionals (e.g., doctors, dentists, pharmacists, etc.), 42.4% are medium-qualified health professionals, and 20.7% are auxiliary health staff. It also shows once again the inequitable distribution of medical staff between rural and urban areas [5]. In 2001, urban healthcare units had 92.1% of all doctors, 89.0% of all dentists, 83.4% of all pharmacists, and 89.4% of all average healthcare staff [5].

In 2021, the healthcare network provided continuous inpatient care for 2,651,230 patients in hospitals, 1458 patients in health centres with hospital beds, 2282 patients in TB sanatoriums, 1037 patients in neuropsychiatric or neuropsychiatric sanatoriums, and 19,713 people in spa sanatoriums [5].

## 3. Health Expenditure in Romania

The public sector dominates healthcare in Romania, owning the majority of hospitals and providing national health insurance to nearly all Romanian citizens [6].

The financing of the health system in Romania always depended on the economic and political transformation the country was going through. In 1946, the budget allocated by the State was 6.42% of the country’s budget, increasing afterwards at quite a steady pace. In the 1950s, the annual growth rate of the total expenditure for health was 22.78%; between 1967 and 1977, the average annual rate of growth of expenditures for healthcare was 7.25%. In the 1980s and until the Romanian revolution in 1989, the variations in the resources allocated for health were considerable, leading to problems in the medical system [7].

After 1989, to reduce expenditure and improve the efficiency of the system, Romania carried out a series of reforms to optimise the hospital infrastructure. In most cases, reform mainly meant closing hospitals or at least reducing their capacity as performance thresholds were introduced and the existing healthcare units were unable to meet them. For Romania, evaluations considered not only the number of readmissions and transfers but also the number of cases that could be avoided, and certain hospitals were considered to underperform, leading to the closure or transformation of 67 hospitals into units of care for elder people. Additionally, hospital networks were put in place for a better allocation of resources and to coordinate the services offered. The network of services provided for a reduction of costs was also achieved in Estonia, Lithuania, and Latvia. Other Eastern Europe countries went through similar processes but with small differences: Hungary preferred to just reduce the number of beds or close sections in certain hospitals, while the Czech Republic centralised specialised care services in different centres and care facilities, increasing the quality by offering specialised treatment just in those places [8].

Still, despite the good intentions of increasing the efficiency of the use of public resources, the long tradition of getting medical care directly from specialists in hospitals created a huge resistance to the idea of reducing the number of people that would get to hospitals for health services by introducing family medicine. It was a needed reform, as the budgets allocated for the health sector were extremely limited [8].

In the 1990s, family medicine started to work, and universities offered new lines of training in the field, generating specialists in family medicine and giving this reform a chance to finally be accepted by the end beneficiaries. At the beginning, the new system was implemented only in a few regions to check its efficiency and allow necessary laws to be passed. Still, there were problems that needed care, such as the lack of control over the quality of services provided as well as over the billing of these services, which seemed to get out of hand. The experience of Romania led to some conclusions regarding the steps to be taken and their order: for the system to work, first the doctors must be trained for their new role in order to quickly gain the trust of future beneficiaries of their services; then, regulations for control and monitoring should be put in place for protection against the misuse of public funds; next come measures regarding the incentives in the payment system and the establishment of private, independent practices for the family doctors, with clear ways of accreditation and ownership over certain primary care facilities that previously belonged to the government [8].

The health reforms in the 1990s dealt with funding sources as well. A special fund for health was created in 1992, and the government provided partial compensation for specific medicines. Contributors to this fund were all employed persons, through a tax on salary, as well as the producers and sellers of alcohol and tobacco, through additional taxation on these types of products. These sources remained the main sources of financing for the next 5 years. After that, besides the compulsory contribution to the health system, private health insurance became an option for the employees [8].

In 1990, the health expenditure was 2.7% of the GDP; in 1998, it was 3.2%; and in 2005, it reached 5.4%. Afterwards, the system financing became more fluctuant, reaching the lowest percentage of GDP in 2015, when the allocation was 4.5% of the GDP, a step back to the value of 2002. In 2017, it went above 5% again, and in 2020, healthcare spending was estimated to be 6.3% of GDP, well below the 10.9% of GDP average for most European Union (EU) countries [9]. The level of current healthcare expenditure was valued at EUR 13.7 billion in 2020. It has grown by 118% in the last decade, reaching EUR 6.2 billion in 2012 [9]. In relation to population size and in EUR, current expenditure on healthcare in 2020 was EUR 713 per capita [9].

## 4. Health Status in Romania

In Romania, one in four people aged 2 and over suffers from at least one chronic disease or long-term health condition [10].

However, in 2021, 72.8% of the Romanian population reported that their health was good or very good; this proportion is close to the average of the European Union countries, with a level of good or very good health of 69.0% [11]. At the other end of the scale, 7.4% rated their health as bad or very bad, and 19.8% rated their health as satisfactory [11]. Perceptions of a bad or very bad health status were more prevalent among the elderly, especially older women.

Life expectancy at birth is 75.0 years, up from 71.2 years in 2000 but still among the lowest in the EU [12].

In 2021, 19.9% of people in Romania aged over 16 years suffered from a chronic disease or long-term health issue, below the EU countries’ average of 35.2% [11].

The infant mortality rate in Romania is 5.26 deaths per 1000 live births [13,14]. This is a significant improvement over the previous years, as the infant mortality rate in Romania has been constantly decreasing over the last decades, being 24.35/1000 live births in 1990, 18.13/1000 live births in 2000, and 10.55/1000 live births in 2010 [13]. The neonatal mortality rate is reported at 3 per 1000 births [15]. Moreover, the under-5 mortality rate is estimated at 6.42 per 1000 live births, with 1281 deaths reported in 2021 [15]. This indicator has also shown remarkable improvements in recent decades, with under-5 mortality being 31.11/1000 live births in 1990, 21.45/1000 live births in 2000, and 12.39/1000 live births in 2010 [15].

The rate of young people suffering from at least one chronic disease is 3.3% in the 0–14 age group, 1.1% in the 15–24 age group, and 4.5% in the 25–34 age group. The older a person is, the more likely they are to suffer from chronic diseases, so the proportion of people aged 55–64 suffering from at least one chronic disease is 43.4%, and the proportion of people aged 65–74 suffering from chronic diseases is 69.1% [10].

The most common chronic diseases in the population aged 15 and over are: hypertension (159 per 1000), low back disease (78 per 1000), diabetes mellitus (50 per 1000), and cervical disease (36 per 1000) [10].

In 2018, ischemic heart disease was the leading cause of death in Romania, accounting for more than 19% of all deaths, followed by stroke (16% of all deaths), and lung cancer (3.9% of all deaths), the latter being the most common cause of death from cancer, with mortality rates increasing by nearly 11% since 2000 [12]. Adult smoking prevalence is currently slightly lower than the EU average. However, while in 2014, 19.8% of adults smoked tobacco every day, today the prevalence of smoking among adults is over 20%, still lower than the EU average [12].

Despite predictions that Romania would have a lower incidence of cancer than the EU average, Romania’s overall cancer mortality was estimated to be slightly higher than the EU average, with 283 deaths per 100,000 people [12].

## 5. Healthcare System in Romania

The healthcare system in Romania is a social health insurance system that has remained highly centralised despite recent efforts to decentralise some regulatory functions. It provides a comprehensive benefits package to 85% of the population, with the remaining population having access to a minimum package of benefits [16].

The healthcare systems comprise all organisations, institutions, and resources that are dedicated to health actions [17].

In the European Union, there are three models of operation and organisation of the public health system: the Bismarck model, the Beveridge model, and the mixed system [18].

Initially, Romania adopted the Semashko model, which happened at the end of 1989, and in the course of time, this model endured several corrections. The reasons why this system did not work for Romania were the conditions under which funding was not optimally administered and the funds that were supposed to flow into the system were almost nonexistent. As a result of these problems, Romania decided to adopt another model, the Bismarck model, adopted in 1997 by Law No. 145, with compulsory health insurance based on the principle of solidarity and operating within a decentralised system.

This model, adopted by Romania, implies that:

- Financial resources are characterised by compulsory contributions that are paid by employees and employers;
- Resources from state, local, or national budget subsidies;
- Non-profit institutions;
- Insurance funds, which are managed and administered at the national level through the social directorates.

The current healthcare system in Romania operates on the basis of Law No. 95/2006, drafted and adopted by parliament, which was subsequently amended in 2012 [19]. Additionally, the primary healthcare law (Act No. 95/2006 on Healthcare Reform) was modified in 2020 to provide the foundation for the advancement and application of telemedicine [20].

The main challenges for the Romanian healthcare system are cost and quality issues. As far as cost issues are concerned, these are related to insufficient funds and inefficiency in the way they are used. In addition, there are also problems related to informal payments, such as money people give to doctors and nurses to get services faster—money that distorts fair access to health services.

The Ministry of Health is in charge of overall social health insurance system governance, while the National Health Insurance House administers and regulates the Single National Health Insurance Fund social health insurance system.

The Ministry of Health is primarily responsible for healthcare in Romania. It is responsible both for the regulatory framework and policies and for the management of the health system in general [21].

The National Health Insurance House (NHIH) administers and regulates the health system. The activity of the National Health Insurance House requires the fulfilment of certain functions. These involve the administration of collected funds and the financing of medical services needed by the insured [22]. The National Health Insurance House is a public, autonomous institution of national interest with legal personality whose main object of activity is to ensure the unified and coordinated functioning of the social health insurance system in Romania.

The NHIH operates on the basis of its own Statute and has the following obligations [23]:

- To ensure the logistics of the unified and coordinated functioning of the social health insurance system;
- To monitor the collection and efficient use of the fund;
- To use appropriate media to represent, inform, and support the interests of the insured persons it represents;
- To meet the health service needs of individuals within the limits of the funds available.

Both the Ministry of Health and the National Health Insurance House have representation at the local level through district public health authorities (DPHAs) and district health insurance houses (DHIHs).

The healthcare system in Romania has undergone reforms, including the reduction of state-funded hospitals and an increase in private investment in medical services. The implementation of healthcare system reforms in Romania has occurred amidst a challenging environment characterised by limited financial and human resources. During the years 1989 to 2001, the series of reforms encompassed the inclusion of social health insurance as well as an amplification of the function of family physicians [24].

A national health strategy between 2014 and 2020 was approved at the end of 2014. The strategy covers the following areas: public health and healthcare (with a focus on improving women’s and children’s health, reducing morbidity and mortality from non-communicable diseases, and ensuring equitable access, especially for vulnerable groups, to quality and cost-effective health services), health research, e-health technologies, and health infrastructure (at the national, regional, and local levels). This strategy aims to protect and improve the health of the population and support the modernisation of health systems [25].

Subsequently, the National Health Strategy 2023–2030 (NHS) was adopted, representing the commitment of the Ministry of Health, as the central authority for developing and coordinating health policies at the national level, to the citizens of Romania in order to improve their healthy life expectancy and quality of life. The current National Health Strategy, “Together for Health”, runs from 2022 to 2030, continues the previous strategy’s objectives, and responds to the need for structural reforms in the health sector. The strategy also serves as the national strategic policy framework for health, within which the fulfilment of the European Commission’s enabling condition for health on the development of the Partnership Agreement and programmes in Romania for the period 2021–2027, as well as the country recommendations issued by the European Commission on the healthcare system, will be assessed [26].

The National Health Strategy 2023–2030 encompasses three key strategic interventions aimed at safeguarding and promoting public health, delivering top-quality healthcare services and technologies that are both safe and accessible, and ensuring the efficiency and coherence of the entire healthcare system [26].

The Romanian health system experienced a significant increase of 14.5% in the number of total units, with the private sector contributing to more than 50% of the growth and establishing over 18,000 new units, as reported by the National Statistics Institute study, which revealed that the private medical system expanded 60 times between 1997 and 2016 and now accounts for 75% of the total, with the number of doctors employed in the private sector increasing to 25.98% in 2014 [27]. Despite a doubling in the number of private hospitals during the analysed timeframe, their overall quantity remained relatively small. They accounted for only 30% of the highest total value recorded in 2015, amidst a decreasing number of public facilities. Private hospitals were able to establish a relationship with the social health insurance system, either fully or partially, after obtaining all necessary authorisations. The vast majority of these facilities are currently being financed by the Single National Fund of Social Insurance (FUNASS) [27].

However, the Romanian healthcare system necessitates an initiative to enhance the calibre of medical services in an effort to augment patient contentment and optimise resource utilisation within the healthcare system [28]. The healthcare system of Romania is predominantly funded by the public sector, accounting for 80.45% of its financial resources. These funds are derived from various sources, namely Social Health Insurance, which contributes 65%, and the State and Local Authorities Budget, which provides 15.45%. Moreover, private sources such as voluntary health insurance and out-of-pocket payments contribute an additional 19.55% to the overall public budget [29]. Expenditure allocated towards hospital services exhibited a portion of 54.7% in the year 2019, in contrast to 55.8% in 1999, showing a significantly stagnant 20 year trend in spite of public proclamations and initiatives aimed at promoting primary care and ambulatory services [29]. Expenditure showing an upward trend included expenditure on medical devices as well as expenditure on medical supplies, dialysis costs, and medical services received in EU expenditure. In the pinnacle year of 2016, the total magnitude of healthcare amenities procured within the European Union amounted to approximately EUR 125 million. The aforementioned sum represents the medical services imported into Romania from providers within the EU. In the time frame from 2008 to 2019, the absolute aggregate quantity disbursed by Romania to furnish medical services obtained within the European Union exceeded EUR 650 million, while over 10,000 physicians emigrated from Romania within the aforementioned duration [29].

The preeminent predicament in the Romanian healthcare system is widely regarded as corruption. An investigation has determined that the primary concern in the Romanian healthcare system is attributed to the perception of corruption, with a majority of those surveyed expressing their opposition to unregulated financial transactions. The overwhelming majority of the study’s participants exhibit opposition towards informal payments and opine that the implementation of co-payments will not inevitably result in a reduction of corruption or informal payments within the health system, nor will it lead to an improvement in the quality of health services if not accompanied by measures that directly target these services [30].

Furthermore, the patients exhibited a general sentiment of discontent with the existing healthcare infrastructure and the practitioners therein. Additionally, the patients held a pessimistic outlook towards the efficacy of the healthcare system.

## 6. Social Health Insurance in Romania

Social health insurance in Romania can be seen as a whole as an insurance programme through which Romanians can insure their health and at the same time finance their medical needs.

Medical services are divided into two categories: those that are paid for by the Health Insurance House and those that are not paid for by the Health Insurance House.

Medical services that are paid for by the Health Insurance Fund [19]:

- Emergency medical services other than those directly financed by the Ministry of Health;
- Medical services provided to the sick person up to the diagnosis of the condition: medical history, clinical examination, and paraclinical investigations;
- Medical and surgical treatment and certain rehabilitation procedures;
- The prescription of the treatment necessary for improvement or cure, including indications concerning living and working conditions, hygiene, and diet.

Insured persons benefit from medicines, with or without personal contribution, on prescription for medicines included in the list of medicines drawn up by the Ministry of Health and NHIH. Insured persons are entitled to receive some home healthcare services, including palliative care at home, and are entitled to medical transport necessary for the performance of a medical service.

The services that are not paid from the fund are:

- Medical services in the event of occupational diseases, accidents at work, and sports accidents, medical care at work, medical care for sportsmen, and sportswomen;
- Certain high-performance medical services;
- Certain dental care services;
- Hotel services with a high degree of comfort;
- Cosmetic corrections carried out on persons over 18 years of age, with the exception of breast reconstruction by endoprosthesis in the case of oncological surgery;
- In vitro fertilisation;
- Medical assistance on request;
- The cost of certain materials necessary for the correction of sight and hearing;
- Personal contribution towards the price of medicines, certain medical services, and medical devices;
- Medical services requested by the insured person;
- Certain rehabilitation services and procedures;
- Family planning services provided by the family doctor in the hospital planning offices.

## 7. Discussion

Current health expenditure per capita refers to expenditure on health goods and services per capita estimated each year. In terms of health expenditure per capita, at the European level in 2018, Germany topped the ranking, spending the equivalent of USD 5472.20 on health goods and services, followed by Finland, which spent the equivalent of USD 4515.68. The UK was in third place, spending the equivalent of USD 4315.43, followed by Spain and Greece. The lowest estimated expenditure in 2018 was made by Romania, equivalent to USD 687.25 per resident [31]. The average per capita expenditure of all 14 other member states in 2018 was EUR 1877 or less, and the average per capita expenditure on healthcare in six of the member states was less than EUR 1000.

In 2020, Switzerland was the biggest spender in Europe, spending EUR 4997 per person, followed by Germany with EUR 4831. In the Netherlands, Austria, and Sweden, spending levels were also well above the EU average [32].

Health expenditure relative to GDP shows how much a country spends on healthcare relative to all other services and goods in the economy and how this fluctuates over time depending not only on the level of health expenditure but also on the size of the economy as a whole (OECD/European Union, 2018). Of the EU member states, in 2018, five spent on average 8.512% on health, with Germany having the highest share of GDP (11.2%). Romania spent the least (5.56%), due to being a country in transition and development. The United Kingdom ranks second with a share of GDP allocated to health spending of 9.8% [31]. In contrast, current expenditure on healthcare was less than 7.5% of GDP in 12 member states, with Luxembourg having the lowest ratio (5.3%) [31].

In terms of current data, in 2021, Germany remains in first place with 12.8% of GDP, followed by France (12.2%), Sweden (11.5%), and Austria (11.5). Romania is still at the bottom of the ranking with 6.3% of GDP, the EU average being 10.9% [32]. Among Eastern European countries, health expenditure relative to GDP is 8.5% in Bulgaria, 7.3% in Hungary, and 6.5% in Poland [9].

Health expenditure measures the final consumption of health goods and services. It includes both public and private expenditure on health goods and services, disease prevention programmes, public health, and per capita administration.

The financial resources that a country allocates to health and care (for individuals or for the population as a whole) and how these fluctuate over time are the result of a wide range of social and economic factors, continuous financing, and the existence of a well-structured organisation of a country’s health system.

In 2019, Germany is estimated to have outperformed all other countries included in the analysis by a wide margin, spending the equivalent of USD 6645.76 per resident. Of the countries covered in the analysis, Romania ranks last in terms of health expenditure per capita in 2019, spending the equivalent of USD 1906.81. In terms of health spending per capita by the United Kingdom, it maintains its position in second place—just behind Germany (as in 2018), spending USD 4653.06 per capita. These sums reflect investment in what is meant by hospital beds in clinics and hospitals; it means investment in specialist and general practitioners as well as nurses (so specialist medical and healthcare staff) [31].

Current expenditure on healthcare in Germany was EUR 384 billion in 2018—the highest among EU member states. France recorded the second highest level of current expenditure on healthcare (EUR 266 billion), followed by Italy (EUR 153 billion) and Spain (EUR 108 billion) [31]. In 2020, Germany also recorded the highest level of current healthcare expenditure among EU member states (EUR 432 billion), followed by France (EUR 281 billion), Italy (EUR 160 billion), and Spain (EUR 120 billion) [9].

Relative to population size, expenditure on healthcare in 2018 was highest among EU member states in Denmark (EUR 5256 per capita), Luxembourg (EUR 5221 per capita), and Sweden (EUR 5041 per capita). Interestingly, Luxembourg has the lowest ratio of healthcare expenditure per capita of GDP and the second highest ratio per capita, reflecting the high level of Luxembourg’s GDP [31]. The latest reports state that current expenditure on healthcare in 2020 was highest among EU member states in Luxembourg (EUR 5875 per capita), followed by Denmark (EUR 5642 per capita). Romania ranks last on this indicator, with EUR 713 per inhabitant. Thus, the ratio between the highest (Luxembourg) and lowest (Romania) levels of expenditure per inhabitant was 8.2:1 [9]. Compared to other Eastern European countries, Hungary has a current health expenditure per inhabitant of EUR 1022, Poland EUR 902, and Bulgaria EUR 754. Among non-EU countries, per capita health expenditure in Ukraine is USD 248 per capita, USD 672 per capita in Serbia, and USD 306 per capita in Moldova [33].

A large share of Luxembourg workers are cross-border workers and live abroad; note that as non-residents, their healthcare costs are not included in Luxembourg’s medical account, and their economic activities contribute to Luxembourg’s GDP.

Liechtenstein, Switzerland, Norway, and Iceland—report higher levels of healthcare expenditure per capita than any of the member states in 2020. Then, in Denmark, Luxembourg, and Sweden, a group of four member states—Germany, Ireland, Austria, and the Netherlands—recorded current expenditure per capita on healthcare between EUR 4480 and EUR 4627. In turn, another group of countries (Belgium, France, and Finland) followed closely behind, with per capita income between EUR 3829 and 4150. The gap with Italy (EUR 2534 per capita), Spain (EUR 2310), and Malta (EUR 2290) is relatively large [31].

National Health Services (NHS) and Social Security-based Healthcare Systems (SSH) are the two broad categories of health system delivery systems [34]. Regarding the health system in Romania, the Bismarck system is adopted. This system is also adopted at European Union level in countries such as the Czech Republic, Belgium, Estonia, Germany, France, Lithuania, Luxembourg, Poland, the Netherlands, Slovakia, Hungary, or Slovenia [18,35]. This model is also known as the social health insurance system, and it is funded by mandatory social insurance contributions made by employers and employees [18,35]. In comparison, in countries such as Cyprus, Denmark, Finland, Ireland, Italy, Latvia, Malta, Portugal, Spain, Sweden, and the United Kingdom, the Beveridge model is adopted [18,35]. This model, which was first implemented in the United Kingdom in 1942, is funded by public taxes, and the state directly finances structures. This model, also known as the National Health Service (NHS), provides universal health coverage.

Because healthcare systems differ and are complex, they are difficult to compare. Various countries and international organisations have investigated methods for assessing the performance of healthcare systems [18]. The World Health Organisation (WHO), the Organisation for Economic Cooperation and Development (OECD), the European Community Health Indicators (ECHI), and Bloomberg L.P., a privately held financial software, data, and media company, have developed the most interesting evaluation models. The WHO index is based on the following factors: life expectancy is adjusted for disability, responsiveness, and a fair financial contribution [18].

## 8. Conclusions

In conclusion, the Romanian healthcare system faces significant challenges but also demonstrates potential for improvement. While the system provides universal healthcare coverage and has made strides in reducing infant mortality rates and improving vaccination rates, it struggles with issues such as inadequate infrastructure, regional disparities, a shortage of healthcare professionals, and limited funding. Addressing these challenges requires a comprehensive approach that focuses on increasing healthcare investments, improving healthcare access in rural areas, enhancing healthcare workforce recruitment and retention, and implementing effective healthcare policies. By prioritising these areas, Romania can work towards building a more robust and equitable healthcare system that ensures quality care for all its citizens.

## Data Availability

Not applicable.
